# Dynamic Copy Number Evolution of X- and Y-Linked Ampliconic Genes in Human Populations

**DOI:** 10.1534/genetics.118.300826

**Published:** 2018-05-16

**Authors:** Elise A. Lucotte, Laurits Skov, Jacob Malte Jensen, Moisès Coll Macià, Kasper Munch, Mikkel H. Schierup

**Affiliations:** Bioinformatic Research Center, Aarhus University, 8000, Denmark

**Keywords:** ampliconic genes, sex chromosomes, copy number variation, human population genetics

## Abstract

Ampliconic genes are multicopy, with the majority found on sex chromosomes and enriched for testis-expressed genes. While ampliconic genes have been associated with the emergence of hybrid incompatibilities, we know little about their copy number distribution and their turnover in human populations. Here, we explore the evolution of human X- and Y-linked ampliconic genes by investigating copy number variation (CNV) and coding variation between populations using the Simons Genome Diversity Project. We develop a method to assess CNVs using the read depth on modified X and Y chromosome targets containing only one repetition of each ampliconic gene. Our results reveal extensive standing variation in copy number both within and between human populations for several ampliconic genes. For the Y chromosome, we can infer multiple independent amplifications and losses of these gene copies even within closely related Y haplogroups, that diversified < 50,000 years ago. Moreover, X- and Y-linked ampliconic genes seem to have a faster amplification dynamic than autosomal multicopy genes. Looking at expression data from another study, we also find that X- and Y-linked ampliconic genes with extensive CNV are significantly more expressed than genes with no CNV during meiotic sex chromosome inactivation (for both X and Y) and postmeiotic sex chromosome repression (for the Y chromosome only). While we cannot rule out that the XY-linked ampliconic genes are evolving neutrally, this study gives insights into the distribution of copy number within human populations and demonstrates an extremely fast turnover in copy number of these regions.

## Abstract (precis)

Ampliconic genes are multicopy genes often located on sex chromosomes and enriched for testis-expressed genes. Here, Lucotte *et al.* developed new bioinformatic approaches to investigate the ampliconic gene copy number and their coding...

AMPLICONIC genes consist of several adjacent duplications of small genomic regions with > 99.9% similarity between copies (Skaletsky *et al.* 2003), and the majority can be found on the sex chromosomes. Their evolutionary turnover is very rapid: only 31% of human X ampliconic genes have an ortholog in mice compared to 95% for single-copy genes (Mueller *et al.* 2013). Most of the human ampliconic genes are protein coding and expressed exclusively in the testis; however, their specific function in gametogenesis is poorly understood.

Recently, we reported that ampliconic genes are significantly associated with X-linked megabase-wide regions of low diversity overlapping between primate species (Dutheil *et al.* 2015; Nam *et al.* 2015), most probably resulting from recurrent selective sweeps. These regions also significantly overlap with genomic areas depleted of Neanderthal ancestry in humans (Dutheil *et al.* 2015), suggesting that they are involved in the emergence of hybrid incompatibilities. This prompted us to suggest that ampliconic genes may be speciation genes in primates (Nam *et al.* 2015).

In mice, a homologous pair of ampliconic genes, *Slx* and *Sly*, located on the X and Y chromosomes, respectively, have been suggested to be involved in hybrid incompatibilities and to have coamplified as a result of intragenomic conflict (Soh *et al.* 2014). Intragenomic conflict, here specifically an arms race between the X and the Y for transmission to the next generation due to meiotic drive, leads to an increased divergence between closely related species and is therefore at the origin of hybrid incompatibilities (Frank 1991; Hurst and Pomiankowski 1991). Indeed, an unbalanced copy number of *Slx* and *Sly* in mice leads to deleterious X–Y dosage disruption in hybrids (Larson *et al.* 2016), and a deficiency in *Slx* provokes a sex ratio distortion in offspring toward males, which is corrected with *Sly* deficiency (Cocquet *et al.* 2012). More generally, misregulation of X and Y chromosome expression during spermatogenesis is pervasive in mice hybrids, affecting meiotic sex chromosome inactivation, a crucial phenomenon during male meiosis (Campbell *et al.* 2013; Larson *et al.* 2016). While the X- and Y-linked genes are repressed during meiosis, postmeiotically, genes in multiple copies have been shown to be expressed in round spermatids, dependent on their copy number (Mueller *et al.* 2008). Similar results were obtained in felids; fertility expression QTL were mapped near X-linked ampliconic genes and sterile hybrids show an overexpression of the X chromosome during meiosis compared to controls (Davis *et al.* 2015), suggesting that this phenomenon is widespread in mammals. While these observations point toward an important role of the ampliconic genes in speciation, little is known about the worldwide distribution of copy number variations (CNVs) in human populations of both X- and Y-linked ampliconic genes, as well as their dynamic of amplification.

Most of the recent studies on ampliconic gene copy numbers have focused on the Y chromosome, and were performed on one human population (62 Danes, Skov *et al.* 2017) or a few individuals from several primate species (Ghenu *et al.* 2016; Oetjens *et al.* 2016). To our knowledge, no population-wide studies on CNVs of X-linked ampliconic genes have been conducted. However, a majority of them are part of the cancer/testis (CT) gene family and have been studied as targets for therapeutic cancer vaccines [see Simpson *et al.* (2005) for a review]. Also, using comparative genomics between human and primate sequences, previous studies reported signals of diversifying selection in CT genes (Stevenson *et al.* 2007; Gjerstorff and Ditzel 2008; Liu *et al.* 2008; Zhao *et al.* 2012; Zhang and Su 2014) as well as recent amplification in the human lineage for the *GAGE* (Gjerstorff and Ditzel 2008; Liu *et al.* 2008) and *CTAGE* families (Zhang and Su 2014).

Therefore, a worldwide-scale description of CNVs in both X- and Y-linked ampliconic genes in human populations is lacking. Mueller *et al.* (2013) have considerably improved the human X chromosome reference genome for ampliconic genes (hg38) using single-haplotype sequencing, thus allowing us to investigate these regions at the population scale, as it was done in Danes for the Y-linked ampliconic genes (Skov *et al.* 2017). Such investigation constitutes a first step in evaluating the importance of these genes in speciation. Indeed, a complete description of the copy number distribution in the human population will shed light on their evolutionary dynamic and their speed of amplification.

Here, we investigate the evolutionary dynamics of ampliconic genes on the X and Y chromosomes in humans to answer the following questions: (i) what is the worldwide copy number distribution of the X- and Y-linked ampliconic genes in human populations, (ii) can we detect signals of nonneutral evolution in the ampliconic gene sequences, (iii) are the XY-linked ampliconic gene dynamics of amplification different from an autosomal set of multicopy genes, and (iv) are ampliconic genes expressed during male meiosis? Thus, we surveyed ampliconic gene CNVs, their coding sequence turnover, and their expression during male meiosis from a published study (Sin *et al.* 2012).

Due to the highly repetitive nature of ampliconic genes, classical methods cannot be used, and we therefore developed new bioinformatic approaches to assess their copy number and nucleotide variability in the Simons Genome Diversity Project (SGDP) data set (Mallick *et al.* 2016), which provides genomic sequencing for 128 human populations including 276 individuals (102 females and 174 males). We report very dynamic copy number evolution suggesting high mutation rates of these regions. Moreover, our results suggest that XY-linked ampliconic genes have a faster turnover of copy number compared to the autosomal multicopy genes included in this study, and that the genes showing high copy number as well as extensive CNVs are expressed during meiotic sex chromosome inactivation (MSCI) and postmeiotic sex chromosome repression (PSCR), two meiotic stages where the sex chromosomes are inactivated and repressed. While we cannot disentangle neutral processes from diversifying selection, this study provides the first global picture of the diversity and turnover of the ampliconic genes in human populations at a worldwide scale.

## Materials and Methods

### Data set

We used the SGDP, panel B and C, which includes 274 individuals—102 females and 172 males—from 128 human populations (Mallick *et al.* 2016). Panel C samples (260 individuals) were processed using a PCR-free library preparation protocol and sequencing protocol. Panel B samples (14 individuals) were processed using a PCR-based library preparation protocol. All samples were submitted to Illumina and 100-bp paired-end sequencing was performed. The median coverage for the whole sample set we used was 41.9 with a minimum of 33.59 and a maximum of 83.23 median genome-wide coverage. The median coverage across regions varied from 39.35 to 45.

Three individuals were removed from the analysis: *S_Palestinian-2*, *S_Naxi-2*, and *S_Jordanian-1*. *S_Naxi-2* was removed because, although this individual is labeled as a male, he has a high heterozygosity on the X chromosome while his Y coverage is similar to males. *S_Palestinian-2* has a low heterozygosity on the X chromosome compared to other females and has an X chromosome coverage comparable to males while having a Y chromosome coverage comparable to females. *S_Jordanian-1* was removed because it is an outlier in all of the copy number analysis. These three individuals had also been removed for some analyses in the SGDP paper for different reasons.

### Identifying the unit of repetition of each ampliconic region

#### X chromosome:

**Figure 1:**
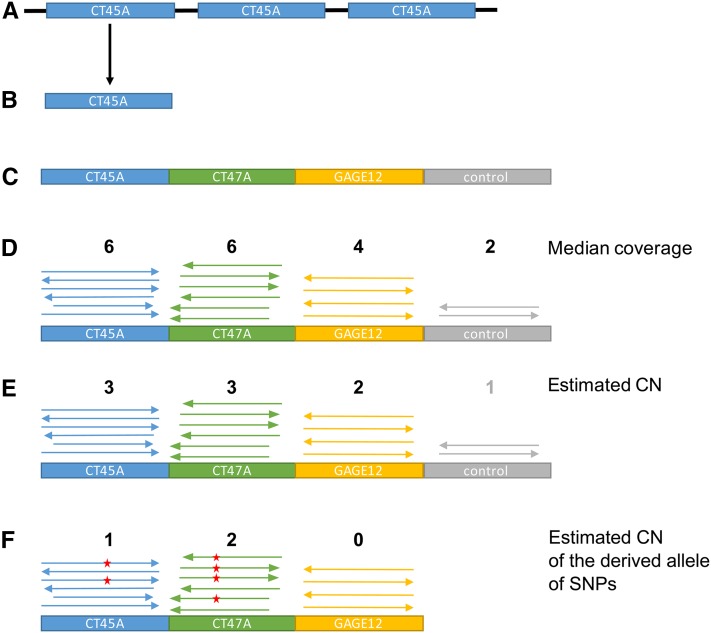
Schema of the artificial X chromosome construction. (A) Identification of the structure of the ampliconic region using dotplots. (B) Selection of one unit of repetition of the ampliconic gene. (C) The sequence of the units of repetition for each ampliconic gene are put next to each other to form an artificial, shorter version of the X chromosome. A control region, known to be single copy, is added. (D) For each individual, the reads are mapped to the artificial X chromosome, and the median coverage of each unit of repetition and the control region is calculated. (E) The median coverage of each unit of repetition is divided by the median coverage of the control region to estimate the copy number (CN) of each ampliconic genes. (F) Variant calling is performed on the alignment, and the CN of the derived allele of each SNP is estimated using coverage.

See Figure 1 for a schema of the method.

The coordinates of the X-linked ampliconic regions were taken from Mueller *et al.* (2013) and were converted to hg38 coordinates. The ampliconic region sequences were extracted from the human reference genome hg38. The sequences of the ampliconic regions were aligned to themselves using *Lastz* (Harris 2007) (–step = 100–notransition–exact = 100–format = rdotplot), and the alignments were filtered to keep only matches longer than 100 bases. We created dotplots to identify the repeated regions.

The same method was used on nonoverlapping 500-kb windows along the X chromosome to identify potential new ampliconic regions and check the existing ones. The boundaries of regions 4, 13, 17, 21, 25, 26, 27, 29, and 32 [taken from Mueller *et al.* (2013)] were enlarged. Regions 10, 14, 17, 18, 30, 31, and 32 were divided into two units of repetition, and region 29 into three units of repetition. One new region was identified (34), containing the genes *CTAG1A*, a CT antigen, and *FAM223A*, a long intergenic nonprotein coding RNA.

We identified the unit of repetition manually on the dotplots. For most of them, the unit was defined by the repeated gene (Supplemental Material, Table S1). For regions that did not contain known genes, we selected the repeated sequence. We identified 24 ampliconic regions on the X chromosome, in which nine are divided into two units of repetition and one is divided into three units of repetition. We performed a BLAST (Basic Local Alignment Search Tool) of the unit sequence against the human reference genome (Altschu *et al.* 1990). Only one region (14_1) showed a significant match with a region on chromosome 9 (98% similarity).

Once the units of repetition were identified, we created mapping targets for raw reads to determine the copy number from read depths. We built an artificial chromosome by concatenating the sequences of each unit of repetition plus an X-linked single-copy gene, *DMD*, for control.

#### Y chromosome:

On the Y chromosome, the ampliconic regions are structurally more complicated than on the X chromosome, because they are organized in palindromes containing several ampliconic genes (Kuroda-Kawaguchi *et al.* 2001; Skaletsky *et al.* 2003). Therefore, we chose to use one copy of each ampliconic gene as a unit of repetition ± 2 kb, as done by Skov *et al.* (2017) (Table S2), instead of searching manually for the repeated sequences with alignments. We also included all coding genes from the male-specific region of the Y chromosome (MSY) for controls. An X-degenerate region on the Y chromosome was used as the control region. Therefore, the artificial Y chromosome is composed of 26 genes, including eight ampliconic genes, and the control region. We also included the sequence of the X chromosome, because most of the Y-linked genes have a closely related X homolog (gametolog). We looked independently at the first two exons of the ampliconic gene *PRY* because it is known that functional copies of *PRY* on palindrome 1 do not have the two first exons, while copies on palindrome 3 do. For the ampliconic gene *RBMY1*, we noticed by looking at the coverage of sliding windows that the end of the gene, which does not contain exons, is not always copied. Therefore, we removed this region from the gene sequence.

### Mapping reads against the short chromosomes

To evaluate the number of copy of each ampliconic region, the read files (fastq files from the SGDP data set) were mapped against the artificial X and Y chromosomes constructed as described above. The read files were also mapped to two control regions: the whole X chromosome excluding the pseudo-autosomal regions (PARs) for the X chromosome, and the X-degenerate region for the Y chromosome. The median coverage for each ampliconic region was corrected by the median coverage of the corresponding control region.

*BWA* 0.7.5 (Li and Durbin 2009) was used to perform the mapping (mem -M -t 16 -a). *sambamba* 0.5.1 (Tarasov *et al.* 2015) was used to filter the paired reads, sort the reads per coordinates, remove the duplicates, and filter the bam files for a mapping quality of ≥ 50 mismatches lower than 2 bp.

For the X chromosome only, we extracted the reads mapped to the artificial X chromosome and remapped them on a reference genome containing the autosomes, the Y chromosome, and the artificial X chromosome. This allowed for reads with a better match on the autosomes to be removed from our analysis. We used the same pipeline than for the first mapping.

After mapping to the artificial chromosomes, the coverage for each position was obtained using *SAMtools* 1.3 (Li *et al.* 2009).

### Variant calling and estimation of the number of copies bearing a variant

A multiple sample variant calling was performed using *platypus* version git-20150612 (Rimmer *et al.* 2014), without filtering, on males and females for the X chromosome and on males for the Y chromosome. The artificial X and Y chromosomes were used as the reference for the variant calling, so the number of reads supporting a variant will be proportional to the number of copies bearing the variant.

The absence of filtering in the variant calling allowed for the inclusion of variants with allelic imbalance, and the copy number of each variant could be assessed by using the read depth. To estimate the number of copies bearing each variant, we multiplied the estimation of the copy number of the gene for each individual by the number of reads supporting the variant, divided by the number of reads covering the variable position.

Variant calling was also performed on a male chimpanzee (M. H. Schierup, C. Hvilsom, T. Marques-Bonet, and T. Mailund, unpublished data) to assess the ancestral allele of the variants called in humans. We mapped the fastq files of the chimpanzee to the artificial X and Y chromosomes constructed with the human reference using the same pipeline as for humans. We filtered for two mismatches in the alignment and for a length of 100 bp. Variant calling was then performed on the alignment using *platypus*. The variant calling in chimp was then confirmed by looking at the base called for each position using the python package *pysam* (https://github.com/pysam-developers/pysam). Both human and chimpanzee vcf files were merged using GATK 3.6 (McKenna *et al.* 2010) and *picard* 2.7.1 (http://broadinstitute.github.io/picard/). No variants were detected in *TSPY*.

### Distance trees

Using the R function *NJ* from the package *APE* (Paradis *et al.* 2004), neighbor-joining trees were constructed on the genetic distances (p-distance) between males for the whole X and the X-degenerate region of the Y chromosome, using SNP data (Nei and Kumar 2000). An alignment was performed on the genotype for each SNP, and a distance matrix was computed for all pairs of individuals. The trees were constructed using the R package *ggtree* (Yu *et al.* 2017).

### Influence of geography and haplogroup on copy number

ANOVA tests were performed in R to assess the influence of the region of origin and haplogroup on copy number. The *P*-values were corrected using the false discovery rate (FDR) method (Benjamini and Hochberg 1995) for multiple testing over the number of ampliconic regions.

### Signature of selection

The exonic variants were annotated and classified as nonsynonymous (NS) (aggregating missense variants, stop gained, or stop lost variants) or synonymous (S) using *SnpEff* 3.6 (Cingolani *et al.* 2012) and the annotation database GRCh38.86. A McDonald–Kreitman test and a direction of selection test were then performed on each ampliconic region and on the pooled ampliconic region to detect signatures of positive selection. Populations were taken into account together and not separately.

### Autosomal ampliconic regions

We selected a sample of 26 autosomal ampliconic genes (Table S3) from: Dennis and Eichler (2016) (10 segmental duplications), Warburton *et al.* (2004) (nine large inverted repeats), and Sudmant *et al.* (2015) (seven genes with CNV in the SGDP). We analyzed these autosomal regions following the same workflow as for the XY-linked regions.

Using dotplots, we identified 17 regions with highly similar copies (Table S4). We then defined the unit of repetition for each region, and the sequence of each unit of repetition was included in an autosomal reference genome along with a control gene, the Lactase gene (*LCT*). We then performed a mapping of the fastq files on this autosomal reference genome for SGDP individuals, following the same pipeline as for the XY-ampliconic genes. The copy number was estimated using the median coverage of the unit of repetition divided by the median coverage of LCT for each individual. We performed a variant calling of the mapping and assessed the copy number of each variant, as done for the XY-ampliconic genes.

### V_ST_

For each ampliconic region, we computed the V_ST_ index on the copy number of the gene and on the copy number of the variants.

$$V_{ST}=\frac{V_{\mathrm{tot}}\;-(V_{A}N_{A}+V_{B}N_{B}+V_{C}N_{C}+\ldots )}{V_{\mathrm{tot}}}$$

where *V*_tot_ is the total variance among all populations, *V_A_* is the variance in copy number in population A, and *N_A_* is the sample size of population A etc.

### Data availability

Data from the SGDP are freely available and have been published by Mallick *et al.* (2016). Supplemental material available at Figshare: https://doi.org/10.25386/genetics.6165824.

## Results

### CNVs of the ampliconic genes

The results for CNVs of all 34 X-linked regions and 27 Y-linked regions can be found in Table S1 and Table S2.

**Figure 2:**
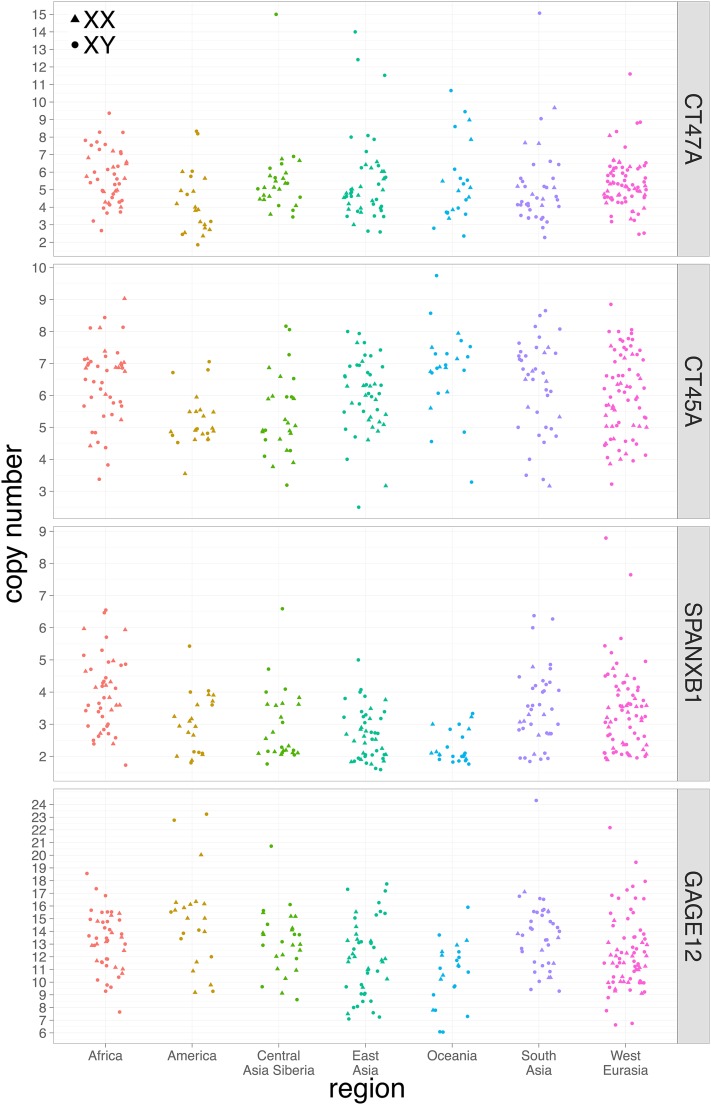
Copy number of X-linked ampliconic genes. Individuals are grouped according to their geographical origin. Females are indicated by circles and males by triangles.

Four out of 34 X-linked ampliconic regions exhibit extensive CNVs: CT ampliconic genes *CT47A*, *CT45A*, *GAGE12*, and *SPANXB1* (Figure 2 and Table 1). The regions 21_0 and 32_0, as referenced in Mueller *et al.* (2013), also harbor extensive CNV but were discarded: the former does not contain any known gene and the latter contains the gene *OPN1LW* (opsin 1, long-wave-sensitive), and we chose to focus on the testis-expressed genes with extensive CNV.

**Table 1:** Summary table of the X-linked ampliconic genes with copy number variation

	Ampliconic region			Unit of duplication				Copy number variation				ANOVA	
AR	Start	End	Length	Gene	Start	End	Length	Min. CN	Max. CN	Δ CN	Med. CN	*q*-value	V_ST_
6_0	49,219,545	49,680,241	460,696	GAGE4	49,560,875	49,568,204	7,329	6.07	24.32	18.25	12.58	**1.09E−04**	0.11
24_0	120,864,869	120,993,348	128,479	CT47A4	120,933,840	120,937,158	3,318	1.86	15.07	13.21	5.01	2.26E**−**01	0.01
26_0	135,656,801	135,914,069	257,268	CT45A5	135,777,129	135,785,475	8,346	2.5	9.75	7.25	6.1	**1.17E−03**	0.08
27_0	140,964,264	141,635,364	671,100	SPANXB1	140,998,306	141,014,744	16,438	1.59	8.79	7.2	3.07	**1.95E−08**	0.16

CN, copy number. Bold values indicate significant *P*-values

**Figure 3:**
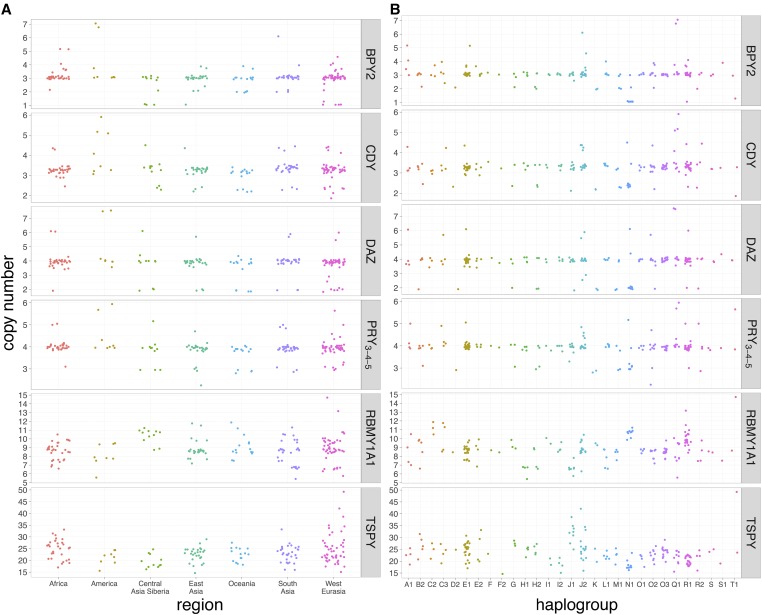
Copy number of Y-linked ampliconic genes. Individuals are grouped according to their (A) geographical origin and (B) Y haplogroup.

On the Y chromosome, six genes harbor extensive CNVs: *BPY2*, *CDY*, *DAZ*, *PRY*, *RMBY1A1*, and *TSPY*, all involved in spermatogenesis (Figure 3, A and B and Table 2), while two genes show minor CNVs: *XKRY* and *HSFY* (Figure S1). Therefore, all the Y-linked genes defined as ampliconic show CNVs, except *VCY*.

**Table 2:** Summary table of the Y-linked ampliconic genes with copy number variation

						COPY NUMBER VARIATION					ANOVA (q-value)		
Gene	Palindromes	Start	End	Length	Min. CN	Max. CN	Δ CN	CN reference	Med. CN	Region	Haplogroup	V_ST_
BPY2	P1 and P2	22,982,263	23,007,465	25,202	1.05	7.06	6.01	**3**	3.05	7.49E−02	**2.94E**−**07**	0.04
CDY	P1 and P5	25,620,115	25,636,745	16,630	1.86	5.9	4.05	**4**	3.28	2.19E−01	**1.67E**−**03**	0.02
DAZ	P1 and P2	23,127,355	23,201,123	73,768	1.82	7.56	5.73	**4**	3.95	1.11E−01	**5.23E**−**03**	0.03
HSFY	P5	18,544,685	18,590,963	46,278	1.94	6	4.06	**2**	2.07	1.38E−01	1.00E+00	0.02
PRY exon1-2	P1	22,482,280	22,492,500	10,220	1.19	3	1.81	**—**	2.15	8.38E−01	1.00E+00	—
PRY exon3-4-5	P3	22,495,000	22,517,543	22,543	2.25	5.94	3.69	**2**	3.95	6.11E−02	**1.05E**−**03**	0.04
RBMY1A1	P3 and IR2	21,532,902	21,550,000	17,098	5.42	14.71	9.29	**6**	8.74	2.29E−01	**6.79E**−**07**	0.03
TSPY	TSPY array	9,464,955	9,471,749	6,794	14.59	49.21	34.62	**35**	23	6.11E−02	**7.58E**−**02**	0.07
XKRY	P5	17,766,980	17,772,560	5,580	1.85	5.9	4.05	**2**	2.06	8.38E−01	1.00E+00	−0.02

CN, copy number. Bold values indicate significant *P*-values

Henceforth, we focused our analysis on four X-linked genes, *i.e.*, *CT47A*, *CT45A*, *GAGE12*, and *SPANXB1*, and six Y-linked genes, *i.e.*, *BPY2*, *CDY*, *DAZ*, *PRY*, *RBMY1A1*, and *TSPY*.

#### Copy number estimation:

We compared the copy number estimations for duplicated sequencing for individuals in SGDP (89 individuals for the X chromosome and 46 individuals for the Y chromosome, Figure S2). The median copy number differences between duplicates are equal to zero for all ampliconic genes. The maximum difference in copy number is 1.66 for *SPANXB1* (region 27_0), 0.29 for *CT45A* (region 26_0), 0.21 for *CT47A* (region 24_0), and 0.37 for *GAGE12* (region 6_0). The differences are lower for the Y chromosome; only *TSPY* and *RBMY1A1* have a maximum difference > 0.1 (0.7 and 0.2, respectively). This analysis suggests that our method for CNV estimation is robust to sequencing experimental error.

For Y chromosomes, the copy number can most often be separated into discrete groups, which suggests that our estimates are good. Moreover, the same method used in Skov *et al.* (2017) for the Y chromosome yielded low error rates in father–son pairs. Yet, such discrete classes are not observed for the X chromosome. This can be explained by a difference in the gene length: most of the Y ampliconic genes are longer than the X ampliconic genes, which could induce more variance in the coverage (Figure S3). We further investigated the relationship between gene length and the accuracy of the estimate on a set of autosomal ampliconic genes that we added in our study. The genes that show the most discrete copy number—*NSFP1*, *GRAP*, *HIC2*, and *ARL17B* (Figure S4)—have the longest length (Figure S3) and are all longer than the X-linked ampliconic genes. Another mechanism that can influence a discrete estimation of copy number is incomplete copies via recombination, that would happen on the autosomes and the X chromosome but not on the Y chromosome. Incomplete copies should manifest themselves through the presence of soft-clipped reads during mapping marking the position of the breakpoints. We undertook a comprehensive analysis of the position of soft-clipped reads in our target X-linked genes but found no evidence for incomplete copies (data not shown).

As a reference control, the copy number of *DMD*, an X-linked gene known to be single copy, was calculated and was between 0.95 and 1.17 copies, with a median at 1.06 copy per chromosome. The median copy number for four Y-linked ampliconic genes corresponds to the copy number of the reference Y chromosome (Table 2). We find differences for the other ampliconic genes: for *PRY* we see two additional copies in our study because we accounted for incomplete copies of *PRY*; for *RBMY1A1* we see two additional copies that are most likely pseudogenes not taken into account in the reference; for *CDY* we see three copies compared to four in the reference, as seen in Skov *et al.* (2017); and for *TSPY* the copy number is very different from the reference, probably because this region is highly variable in copy number.

#### Copy number dynamics between populations:

Individuals were separated into geographical groups of origin (Figure 2 and Figure 3A). For both X- and Y-linked ampliconic genes, CNV is extensive within geographical groups, the most extreme example on the X chromosome being *GAGE12*, for which copy number ranges from 6 to 22 in West Eurasia, and the most extreme example on the Y chromosome being *TSPY*, from 15 to 50 copies in West Eurasia. Differences in copy number can be observed between geographical groups. The effect of geographical groups on copy number is significant after correction for multiple testing for three out of four X-linked genes studied: *CT45A*, *GAGE12*, and *SPANXB1* (ANOVA, FDR *P*-value < 0.05). However, the influence of geography on copy number does not seem to follow any obvious geographical pattern on a map (Figure S5). While none of the Y-linked ampliconic genes show a significant effect of geography on CNV (ANOVA FDR *P*-value < 0.05, Figure S6), they all show a significant effect of haplogroups after correction for multiple testing (Figure 3B, ANOVA FDR *P*-value < 0.05). However, CNV is repeatedly found within most haplogroups (Figure 3B) and distant haplogroups can harbor the same number of copies, while closely related haplogroups harbor different copy numbers. We computed the V_ST_ index for each gene, an equivalent of F_ST_ calculated with copy number instead of allelic frequencies. V_ST_ is an estimator of the amount of CNV that is explained by the region of origin. The X- and Y-linked values of V_ST_ are all < 0.2 (Figure S7), which is a cut-off used to determine significant copy number differences between populations (Sudmant *et al.* 2010, 2015).

We also observed large-scale variants affecting complete palindromes on the Y chromosome (Figure S8). For example, in America, we see that two individuals from the Zapotec population have a duplication of palindrome 1 and 2, *i.e.*, eight copies of *DAZ* (+ 4 copies compared to the reference), seven copies of *BPY* (+ 4), four copies of *PRY_3-4-5_* (exons 3, 4, and 5; + 2), and five copies of *CDY* (+ 2). The individual *S_Karitiana-1* has one complete and one incomplete duplication of palindrome 5, *i.e.*, six *HSFY* (+ 4), six *CDY* (+ 3), and six *XKRY* (+ 4). In West Eurasia, two individuals (*S_Adygei-1* and *S_Tuscan-2*) have a duplication of palindrome 2, *i.e.*, six *DAZ* (+ 2) and four *BPY2* (+ 1).

The correlation between copy number of every possible pair of ampliconic genes on the X and on the Y chromosome was calculated, both with all populations taken together and separately. After correction for multiple testing (FDR), no significant correlation was detected.

### Coding variation within the ampliconic genes

We performed single-nucleotide variant calling on the aggregated copies of the ampliconic genes, using the alignment of the reads on the modified X and Y chromosomes. We allowed for allelic imbalance since a given variant may only be polymorphic in some of the copies, but our approach does not allow us to tell which copy the variant is located in. Each variant was annotated as intergenic, S, or NS (Table 3, individual gene results shown in Table 4, Table S1, and Table S2). We used a chimpanzee sequence as an outgroup to estimate the ancestral allele of each variant.

**Table 3:** Number of polymorphic and fixed SNPs per gene, and results of the degree of selection and McDonald–Kreitman test (α)

	Polymorphic		Fixed				
Gene	NS	S	NS	S	α	DoS	*P*-value
GAGE4	7	2	4	0	1.00	0.22	1.00
CT47A4	19	7	6	3	−0.36	−0.06	0.69
CT45A5	10	4	4	1	0.38	0.09	1.00
SPANXB1	6	2	2	1	−0.50	−0.08	1.00
BPY2	3	0	2	2	NA	−0.50	4.29E−01
CDY	14	6	14	7	−0.17	−0.03	1.00E+00
DAZ	0	0	0	0	NA	NA	1.00E+00
HSFY	3	1	9	1	0.67	0.15	5.05E−01
PRY all exons	0	5	6	0	1.00	1.00	2.16E−03
RBMY1A1	23	10	20	7	0.20	0.04	7.79E−01
XKRY	0	0	0	0	NA	NA	1.00E+00

NS, nonsynonymous; S, synonymous; DoS, degree of selection; NA, not applicable.

**Table 4:** Number of polymorphic and fixed SNPs in the X- and Y-ampliconic genes

	Polymorphic		Fixed			
	NS	S	NS	S	α	DoS
X (all genes)	223	111	114	40	0.29	0.07
Y (all genes)	73	42	143	59	0.28	0.073

NS, nonsynonymous; S, synonymous; α, McDonald–Kreitman test; DoS, degree of selection.

A McDonald–Kreitman test and a direction of selection test were both performed on each ampliconic gene and on the joint set of genes, on all populations put together. There is no significant evidence for adaptive evolution (Table 3 for the ampliconic genes showing major CNV and Table 4 for the pooled genes).

Next, for the subset of ampliconic genes with ample CNVs defined above, we investigated the number of copies carrying each derived nonsingleton variant in relation to the copy number of the gene (see Figure S9 for a schematic of the method). We separated common variants, *i.e.*, present in at least two copies in at least one individual (Figures S10–S13), and rare variants, *i.e.*, present in more than two copies.

Due to the multicopy nature of ampliconic genes, it is not possible to derive a classical site frequency spectrum for variants. Instead, for the genes selected in this study, we calculated the number of copies bearing the derived allele of each variant in the whole sample and compared it to the summed number of copies of the genes in the whole sample (Table 5). For the X chromosome, we find that one of the 16 S variants has a frequency > 10%, whereas this is the case for 6 out of 40 NS variants. For the Y chromosome, 15 out of 39 NS variants are have a frequency > 10%, compared to 4 out of 21 S variants.

**Table 5:** Site frequency spectrum of the NS and S variants

	X-linked variants		Y-linked variants	
Frequency	NS	S	NS	S
0	34	15	24	17
0.1	2	0	5	0
0.2	0	0	2	2
0.3	1	0	4	0
0.4	0	1	1	0
0.5	0	0	1	2
0.6	0	0	1	0
0.7	1	0	1	0
0.8	1	0	0	0
0.9	1	0	0	0

NS, nonsynonymous; S, synonymous.

**Figure 4:**
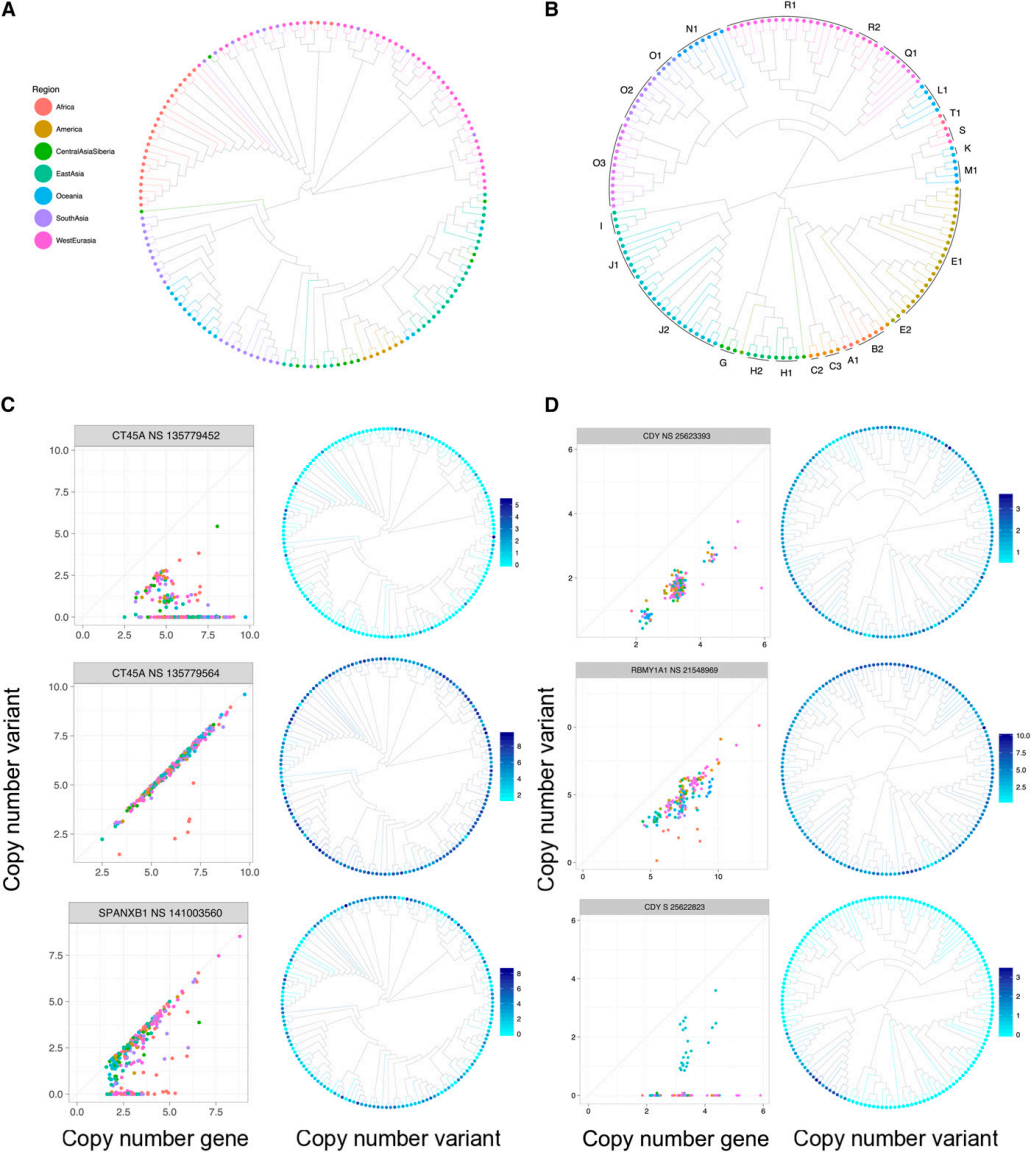
Distribution of copy numbers bearing the derived allele of variants. Tree of the neighbor-joining distance between males for (A) the X chromosome colored according to the geographical origin of each individual and (B) the X-degenerate region of the Y chromosome colored according to the haplogroup of each individual. Number of copies of the derived alleles compared to the number of copy of the gene for each individual for three representative example of (C) X-linked variants and (D) Y-linked variants. The variants are called according to the gene they are located in, whether they are synonymous (S) or nonsynonymous (NS), and their genomic positions. Next to these plots are the distance trees colored according to the copy number of the derived allele of each individual for the variant represented on the left.

We then focused on common variants to assess the amplification dynamics. Most of the variants that are present in more than two copies are widespread across populations, with no clear geographical pattern of amplification (Figures S10–S14, see Figure 4 for examples). Overall, individuals with similar copy numbers of a gene can carry different copy numbers of a variant, suggesting either independent copy number amplification or extensive gene conversion between copies.

To further assess how fast amplification and loss of copies occurred, we constructed neighbor-joining trees with the genetic distance between males for the whole X chromosome and the X-degenerate region of the Y chromosome using SNPs, and colored the leaves according to the copy number of each variant present in more than two copies (Figure S15). Globally, we can see that individuals that are closely related do not necessarily bear the same copy number of a variant. Here, we focused on three representative examples of variants for the X and the Y chromosomes, shown in Figure 4. For the Y chromosome, because it does not recombine outside of the pseudoautosomal regions, we can infer multiple events of loss and amplifications of copies bearing some variants (*CDY* NS 25623392 and *RBMY1A1* NS 21548968), while *CDY* S 25622822 represents a simple case of emergence and amplification of a variant in one branch. For the X chromosome, we observe the same pattern as for most of the Y chromosome variants: individuals that are closely related do not have the same copy number of the variant.

Additional analyses of the genetic distance trees of the X-linked ampliconic genes flanking regions show that the copy number of the variants can be explained by genetic distances, while it is not the case for the copy number of the gene (data not shown), thus indicating independent and very rapid amplification of the X-linked ampliconic genes.

### Comparison with the autosomes

We selected a sample of 26 autosomal ampliconic genes (Table S3) to compare the amplification dynamics with the sex chromosome ampliconic genes. Seventeen regions showed enough similarity between their copies to be included in the study, of which 12 showed significant CNV (Figure S4 and Table S4).

First, we calculated the V_ST_ index for these regions. We compared the distribution of V_ST_ for autosomal genes with significant CNV with the sex chromosome ampliconic regions included in our study. Our results show that the distribution of V_ST_ for autosomes is not significantly higher than for the X chromosome, but is significantly higher than for the Y chromosome (Figure S16, Wilcoxon test, *P*-value = 0.03). This indicates that the autosomal and the X chromosome ampliconic genes display the same stratification of copy number per population. However, for the Y chromosome, the stratification in populations explains less variation in copy number than both X-linked and autosomal ampliconic regions. We also computed V_ST_ for the copy number of NS and S variants (Figure S17). Autosomal V_ST_ values are significantly higher than X and Y chromosome V_ST_ values for NS variants. This suggests that the number of copies of an X or Y-linked variant is less population-specific than for the autosomes.

Second, we compared the median copy number to the variance in copy number for each of the ampliconic genes that showed significant CNV, as done in Ye *et al.* (2018) (Figure S18). Ampliconic genes with the largest number of copies are also more variable in copy number. The X- and Y-linked ampliconic genes are in the tail of the distribution compared to autosomal genes, which suggests that they have a higher number of copies and a higher variance in copy number than the autosomal genes (Figure S18). We performed the same analysis for the NS and S variants. For NS variants, the X chromosome is at the upper tail of the distribution compared to autosomes and most of the Y chromosome variants are at the lower tail of the distribution (Figure S19).

### Expression during meiosis

**Figure 5:**
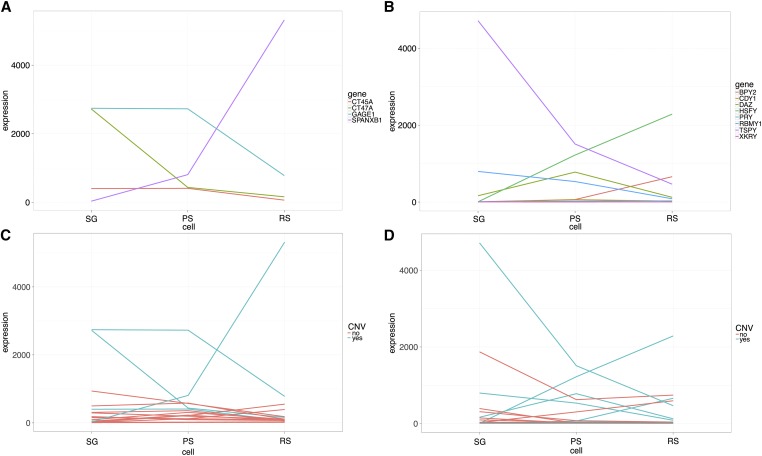
Levels of expression during different stages of male meiosis of (A) X-linked ampliconic genes and (B) Y-linked ampliconic genes with extensive copy number variation (CNV); (C) all X-linked genes and (D) all Y-linked tested genes included in our study. For (C and D), blue lines correspond to the expression of ampliconic genes with extensive CNV and red lines correspond to the expression of the other genes included in our study. On the *x*-axis, different cell types are represented: spermatogonia (SG) before meiosis, pachytene spermatocytes (PS) during meiosis and meiotic sex chromosome inactivation, and round spermatids (RS) during postmeiotic sex chromosome repression. Expression data were taken from Sin *et al.* (2012).

Using the data from Sin *et al.* (2012), we looked at the expression of the ampliconic genes showing major CNV on the X chromosome (*CT45A*, *CT47A*, *GAGE1*, and *SPANXB1* gene families, Figure 5A) and on the Y chromosome (*BPY2*, *CDY1*, *DAZ*, *HSFY*, *PRY*, *RBMY1*, *TSPY*, and *XKRY* gene families, Figure 5B) in humans, and compared it to the other genes included in our study.

These data inform us about the level of expression in three cell types with increasing differentiation during spermatogenesis: spermatogonia before meiosis, pachytene spermatocytes during meiosis and MSCI, and round spermatids during PSCR.

We find that the X-linked genes showing high CNVs are all expressed in pachytene spermatocytes during MSCI, and that two of them are expressed during PSCR (*SPANXB1* and *GAGE1*). The *SPANXB1* gene family is the most striking example, with an expression that increases through meiosis and more dramatically during PSCR. Four of the Y-linked ampliconic genes showing high CNV are expressed during MSCI in pachytene spermatocytes (the *TSPY*, *HSFY*, *DAZ*, and *RBMY1* families), and three gene families are expressed in round spermatids during PSCR (*TSPY*, *BPY2*, and *HSFY*). Moreover, *BPY2* is expressed only during PSCR, which indicates that it is inactivated during MSCI and reactivated during PSCR.

Strikingly, genes that harbor extensive CNV show a significantly higher expression in pachytene spermatocytes during meiosis and MSCI for both X and Y chromosome genes, and a significantly higher expression in round spermatids during PSCR for Y chromosome genes, as compared to genes that do not show extensive CNV (Figure 5, C and D).

## Discussion

Four X-linked ampliconic genes (*CT47A*, *CT45A*, *GAGE12*, and *SPANXB1*) and six Y-linked ampliconic genes (*BPY2*, *CDY*, *DAZ*, *PRY*, *RMBY1A1*, and *TSPY*) harbor extensive CNVs within and among human populations. For the Y chromosome ampliconic genes, the concordance between our results and previously published studies, as well as individuals consistently clustering around discrete values, indicate that copy number estimations are accurate. The X chromosome copy number estimations are less discrete, which can be attributed to their smaller gene length introducing uncertainty in the estimations. However, our method is robust to duplicate sequencing, which is a good indicator that sequencing technology does not introduce variance in our estimates.

While geography/haplogroup has a significant effect on X/Y ampliconic gene copy numbers, the V_ST_ values calculated for these genes are low, which indicates that the overall population structure is weak. For the X chromosome, because of recombination, we cannot conclude that changes in copy number happened independently between populations. However, for the Y chromosome, amplification and loss of copies happened repeatedly on different branches of the Y haplogroup phylogeny, suggesting that changes in copy number occur on a faster scale than the diversification of haplogroups, which mainly occurred within the past 60,000 years (Jobling and Tyler-Smith 2017). This is consistent with previous studies on Y chromosome CNVs (Repping *et al.* 2006; Johansson *et al.* 2015; Wei *et al.* 2015; Poznik *et al.* 2016; Skov *et al.* 2017). Although the process of amplification and loss of copies is very dynamic, copy numbers are kept within a relatively limited range in all populations and are not subject to a runaway amplification process. The absence of correlation between X- and Y-linked ampliconic gene copy numbers indicates that there is not a simple pattern of coamplification between the X- and Y-linked ampliconic genes, like seen in mice.

The ampliconic genes are protein coding and some of the X-ampliconic genes have previously been reported to be under adaptive evolution in primates (Stevenson *et al.* 2007; Liu *et al.* 2008; Zhang and Su 2014). However, in our study, there is no significant evidence of adaptive evolution for any X- or Y-linked ampliconic gene. Thus, we cannot rule out that these genes are evolving under relaxed purifying selection against amino acid substitutions. However, these selection tests do not take the number of copies of variants into account, which could be crucial as the number of copies might affect the level of expression of the gene. Conversely, the site frequency spectrum suggests that the gene conversion and copy number processes preferentially promote the spread of new NS variants both among individuals and among gene copies. Moreover, in the ampliconic genes with extensive CNV, we detected more NS variants than S variants in the ampliconic genes studied here: 67% of the variants are NS for the X ampliconic genes and 63% of the variants are NS for the Y ampliconic genes. This observation could be explained by selective forces driving a rapid differentiation of these regions or by a neutral process that happens faster because of the ampliconic nature of these regions.

We then investigated the amplification dynamics of NS and S variants. For both X- and Y-linked ampliconic genes, the variant copy numbers are not consistent with the genetic distance tree. Individuals with the same Y haplogroup can bear different copy numbers of the derived allele of variants, which is concordant with amplifications and losses happening after the differentiation with their common ancestor, between 60 and 30 KYA. We can conclude that independent losses and amplifications of ampliconic genes have happened and, importantly, that the events happened since the diversification of the Y haplogroups, which suggests that this process is extremely fast. For the X chromosome, because of recombination, we cannot infer that these events are independent based on whole X distance trees; however, an analysis of the genetic distance trees in the flanking regions of X-linked ampliconic genes suggests that amplification is also happening independently. It seems that diversity has been kept within populations for these ampliconic regions, both in terms of copy numbers of genes and variants.

The discrepancies that we observe between individuals from the same population could be due to complex selective events due to X–Y conflict happening not on copy number, but on combinations of copy number and allele-matching processes. High diversity seems to have been kept within populations, which might suggest balancing selection on both Y and X chromosome ampliconic genes. However, the analyses performed in this study cannot reject the possibility that these regions are evolving under relaxed purifying selection.

We then compared several characteristics of sex-linked ampliconic genes with a set of autosomal ampliconic genes. First, the sequence divergence between copies is higher for X and Y ampliconic genes compared to the autosomes, indicating that the X- and Y-linked ampliconic regions are evolving under more relaxed purifying selection or under diversifying selection. Second, we compared the V_ST_ index, which quantifies the amount of variance in copy number explained by the different populations. The autosomal population differentiation in copy number is similar to that of X-linked ampliconic genes, and is significantly higher than for the Y chromosome. Therefore, the Y-linked ampliconic genes have a faster turnover of copy number than autosomes, which is unexpected due to the absence of recombination. Additionally, the V_ST_ for NS variants is lower for the X and Y chromosomes compared to the autosomes, which indicates a faster amplification process for both X and Y ampliconic genes. Third, we compared the relationship between the median copy number and the variance in copy number. Ampliconic genes with a higher median copy number tend to have a higher variance in copy number. This observation is expected, because the probability of change in copy number increases with the number of copies (Ghenu *et al.* 2016). The X and Y ampliconic genes are overrepresented at the top of this distribution; they have a higher median copy number and variance than the autosomal ampliconic genes.

Overall, our analyses suggest a faster turnover of copy number for sex chromosome ampliconic genes compared to autosomal ampliconic genes.

The sex chromosomes are inactivated at the end of meiosis, during pachytene and diplotene (MSCI), and remain repressed during spermiogenesis (PSCR). However, some genes can escape this process and still be expressed during these stages (Sin *et al.* 2012). It has been suggested that amplification allows genes with an important function in spermatogenesis to increase their expression and therefore counterbalance the repressive effect of MSCI and PSCR. Using the data produced by Sin *et al.* (2012), we show that XY-linked ampliconic genes that have high CNV are significantly more expressed during MSCI (for both X and Y) and PSCR (for the Y only) than other XY-ampliconic genes. Using an independent RNA sequencing (RNAseq) data set from Lesch *et al.* (2016), we also found that ampliconic genes showing CNV are expressed in pachytene spermatocytes and round spermatids, except for *BPY2* (see Figure S20, Figure S21, and supplemental text). The differences in expression between genes showing CNV and no CNV was significant for the X-linked ampliconic genes but not for the Y-linked ampliconic genes. We found variable expression levels between the three human samples, which could be due to different copy numbers of the genes, although we cannot test this hypothesis on the RNAseq data set.

Sin *et al.* (2012) showed that escape genes in humans are often *de novo* genes that appeared in the primate or great ape lineage, and that they show a higher Ka/Ks ratio than nonescape genes and therefore undergo a faster rate of evolution. This is concordant with the dynamic copy number gain/loss and the high mutation rate of the ampliconic genes highlighted in our study.

The ampliconic genes with extensive CNV are expressed specifically in testis during meiosis and are good candidates for hybrid incompatibility emergence. Interestingly, in humans, regions depleted of Neanderthal and Denisovan ancestry are enriched for genes expressed in the testis (Sankararaman *et al.* 2016), and particularly in genes expressed during meiosis (Jégou *et al.* 2017).

Further studies on the impact of CNVs and the copy numbers of variants on gene expression and sperm phenotypes will allow us to assess the role of the ampliconic genes in hybrid incompatibility emergence.
